# Takotsubo cardiomyopathy in a female presenting with status asthmaticus: a case report and review of literature

**DOI:** 10.1186/s43044-022-00310-9

**Published:** 2022-10-01

**Authors:** Lindsey C. Clark, Arjun Khunger, Walif Aji

**Affiliations:** 1 Department of Internal Medicine, Memorial Hospital West, GME Building, 703 North Flamingo Road, Pembroke Pines, FL 33028 USA; 2 Department of Cardiology, Memorial Hospital West, Pembroke Pines, FL USA

**Keywords:** Takotsubo syndrome, Takotsubo cardiomyopathy, Cardiomyopathy, Status asthmaticus, Case report, Review of literature

## Abstract

**Background:**

Takotsubo cardiomyopathy (TCM) is a non-ischemic syndrome characterized by transient acute left ventricular dysfunction as evident on transthoracic echocardiography. It can often mimic myocardial ischemia and is characterized by the absence of angiographic evidence of obstructive coronary artery disease. Reports of Takotsubo syndrome in elderly with asthma exacerbations have been noted.

**Case presentation:**

We describe a case of TCM in a 68-year-old female who presented with acute shortness of breath secondary to status asthmaticus. Her electrocardiogram showed ST segment elevations in multiple coronary artery distributions and mildly elevated troponin levels. Coronary angiography showed no significant stenosis of the coronary arteries with left ventriculography that showed systolic apical ballooning with a 10% ejection fraction, consistent with TCM.

**Conclusions:**

Takotsubo syndrome should be considered in the differential diagnosis of patients presenting with status asthmaticus and elevated troponin levels on admission. Patients should be asked about the use of beta agonist prior to admission. A thorough literature review including a summary of 11 previously published case reports of TCM with acute asthma exacerbations has been presented.

**Supplementary Information:**

The online version contains supplementary material available at 10.1186/s43044-022-00310-9.

## Background

Takotsubo cardiomyopathy (TCM) is an acute cardiac dysfunction that typically represents hypokinesis of the apical segment of the left ventricle (LV) beyond a single coronary artery territory [1]. It presents with typical features of myocardial ischemia including central tightening chest pain and shortness of breath, electrocardiographic changes which mimic coronary artery disease, and minimal release of myocardial enzymes in the absence of angiographically significant coronary artery stenosis. The condition is relatively rare and is found in about 1–2% of all patients with suspected acute coronary syndromes (ACS) [2]. It is commonly seen among post-menopausal women [3]. The underlying pathophysiology of this acute cardiac entity remains largely unclear, but is often associated with physical or emotional stress, hence the term “stress cardiomyopathy” [4]. Catecholamine surge-induced cardiomyocyte injury in response to emotional or physical stress has been postulated as its primary pathophysiology [5]. The list of potential triggers associated with TCM continues to expand as the disease gains increasing recognition among internists and cardiologists. The condition is usually benign, and reversible, with nearly full recovery expected in around 6–8 weeks [6]. Rarely, it may be complicated by life-threatening sequelae including acute cardiogenic shock, lethal ventricular arrhythmias, or ventricular wall rupture [7]. In this report, we present a case of TCM secondary to asthma exacerbation along with a review of the published case reports (Table 1) [8–18]. This report emphasizes the importance of the awareness of the potential association between status asthmaticus and TCM.

**Table 1:** Literature review of cases of Takotsubo cardiomyopathy in patients with asthma exacerbation

Study	Age/Gender	Presentation	EKG changes	Peak Troponin levels	BNP levels	Management	Outcome	Possible etiology/preceding stressor
Kansara et al. [8]	58 years/male	Dyspnea, chest pain, wheezing, psychiatric exacerbation	New RBBB plus Left anterior fasicular block	4.9 ng/ml	*Not given*	Initial ECHO normal, Repeat ECHO showed LV Apical Ballooning, Patient refused cardiac catheterization	Repeat ECHO 8 weeks later showed resolution of WMA	Agitation due to psychiatric disturbance/asthma exacerbation
Kotsiou et al. [9]	43 years/female	Chest tightness, dyspnea, dry cough, Salbutamol use 3 times a day prior to admission Stressful family event the day before	TWI in II, III, AVF	2.2 ng/ml	345 pg/mL	Nebulized bronchodilators, IV steroids, adrenaline given. Pt intubated; repeated bronchodilators, IV steroids, and Magnesium Sulfate; ECHO showed 45% EF and Apical ballooning Repeat ECHO showed recovery of LV WMA. Cardiac catheterization showed normal coronaries, EF 60%, no WMA	Repeat EKG 2 months after discharge was normal	Epinephrine use, beta agonist in treatment of Status asthmaticus
Ozturk et al. [10]	58 years/female	Dyspnea, chest pain, wheezing	Diffuse ST depression, Precordial TWI	0.672 ng/ml	*Not given*	ECHO showed hypokinesis of mid/apical segment of intraventricular septum, LV anteroseptal wall, and hyperkinesia of the basal segment, EF 35% Cardiac catheterization revealed normal coronaries, hypokinesis of LV except bases and apex of LV	Repeat ECHO showed normal EF and no segmental WMA	Physiological stress of asthma exacerbation
Khwaja et al. [11]	51 years/female	Dyspnea, wheezing; hospitalized for asthma exacerbation 12 days prior	ST elevation in precordial leads + TWI in inferior leads	5.557 ng/ml	9490 pg/mL	Salbutamol/ipratropium nebulizer and IV steroids, IV aminophylline, antibiotics, BiPAP. Cardiac catheterization showed normal coronaries, EF 30% and apical akinesia and basal segment hyperkinesia	Repeat ECHO showed normal LV systolic function and no segmental WMA	Methylxanthines increase Norepinephrine release and trigger negative inotropic response by way of G-protein signaling
Saito et al. [12]	63 years/male	Dyspnea, wheezing	ST elevation V2-V6 With TWI in II, III, AVF, V2-V6	3.45 ng/ml	703.3 pg/ml	Non-invasive ventilation, IV steroids, continuous SABA nebulizer and inhaled anticholinergic. Cardiac Catheterization showed normal coronaries, EF of 49%, and Apical Ballooning	Repeat EKG normal, ECHO with normal EF	LABA Overdose, stress of asthma attack
Marmoush et al. [13]	80 years/Female	Dyspnea, wheezing, left-sided substernal chest pain	New LBBB	1.112 ng/ml	*Not given*	IV steroids, albuterol/ipratropium plus Aspirin, ECHO showed EF 65% with hypokinesis of LV apex and distal septum. Cardiac catheterization showed apical ballooning	Persistent LBBB; repeat ECHO showed normalized EF, resolution of Apical WMA	Increasing beta agonists use in mild asthma exacerbation
Salahudin et al. [14]	50 years/male	Acute respiratory failure requiring mechanical ventilation	ST elevation in precordial leads	2.29 n/ml	*Not given*	ECHO showed EF 25–30%, with cardiac catheterization showing normal coronaries, apical dilation and balooning.	Repeat ECHO showed normal EF and no apical ballooning	Albuterol (total of 50 gm of albuterol daily in the preceding 24 h) plus stress of asthma exacerbation
Pontillo et al. [15]	72 years/male	Dyspnea	ST Elevation in anterior leads	Fourfold rise in troponin (*values not given)*	*Not given*	ECHO showing apical ballooning and EF 37%	Repeat ECHO showing normal cardiac function	Physiological stress of Asthma exacerbation
Rennyson et al. [16]	66-year old/female	Dyspnea; hypoxia, substernal chest pain	ST Elevation in V1-V4	Initial—normal, second mildly elevated (values not given)	*Not given*	Emergent cardiac catheterization which showed normal coronaries/EF 15%	Repeat admission 6 months later with same complaints and cardiac findings	High dose beta agonists with continued use, with repeat presentation again at 6 months
Stanojevic et al. [17]	71 years/female	Worsening dyspnea requiring mechanical ventilation	Mild ST Elevation in V2–V3 and prolonged corrected QTc	2.6 ng/ml	*Not given*	ECHO showed EF of 35% with severe hypokinesis of basal segments; refused cardiac catheterization	4-weeks later EF of 55% and complete resolution of the RWMA	Excessive albuterol use for worsening asthma 5 days prior to admission
Osuorji et al. [18]	46 years/female	Worsening dyspnea requiring mechanical ventilation	ST elevation in inferior and lateral leads	9.56 ng/ml	*Not given*	Received ketamine and epinephrine to treat bronchoconstriction and developed ST Elevation; Coronaries normal; placed on IABP	Repeat ECHO 3 days later showed normal EF (55%) (Initial EF 10%)	IV epinephrine and ketamine use and status asthmaticus
This study	68 years/female	Dyspnea for 3 days requiring BiPAP, sputum production	LBBB	9.55 ng/mL	20,242 pg/mL	ECHO showed EF 24%, severely depressed LV function, no RWMA Cardiac Catheterization showed EF 10%, LV, normal coronaries, akinesis of anterior/inferior wall and apex; IABP placed	Repeat ECHO 9 weeks showing normal EF	Status asthmaticus

## Case presentation

A 68-year-old female presented to the Emergency Department with chief complaint of shortness of breath, wheezing, and cough for past 3 days. Prior to the hospital visit, she was seen by her primary care provider who prescribed her clarithromycin and Medrol dose pack but she had no significant benefit. She denied any episodes of chest pain or fever.

Her medical history was significant for asthma since childhood, hypertension, hypothyroidism, and left lower extremity deep venous thrombosis 15 years back. The patient reported an eight-pack-year smoking history. There was no personal history of coronary artery disease, prior myocardial infarction, or hyperlipidemia. She had no history of unusual emotional stress prior to admission.

**Fig. 1:**
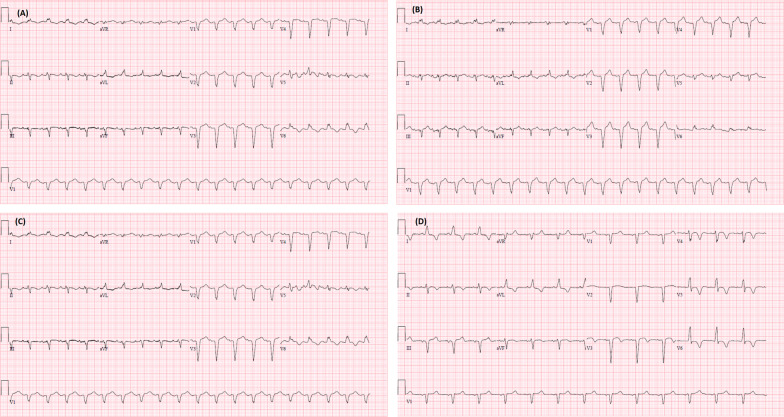
EKG changes during the course of admission. **A** EKG on admission showing ST elevation in inferior and anterolateral leads. **B** EKG 1.5 h later showing same ST elevation in the inferior and anteriolateral leads, and left axis deviation. **C** EKG 18 h later showing new left bundle branch block. **D** EKG 6 days after admission showing resolution of Left Bundle Branch Block

On examination, patient was afebrile with temperature of 36.3 °C, heart rate of 116 beats/minute, blood pressure of 102/75 mm Hg, and respiratory rate of 22 breaths/minute. Bilateral wheezes were heard on chest auscultation. In the emergency room, patient was placed on bilevel positive airway pressure (BiPAP) to maintain oxygen saturation. Appropriate management for asthma exacerbation was started and patient received methylprednisolone 125 mg IV one time along with repeated albuterol nebulizers, Ipratropium–albuterol inhalers, and magnesium sulfate. Initial Chest radiograph revealed mild flattening of diaphragm without any acute process, cardiomegaly or effusions. Electrocardiogram (EKG) on admission showed mild ST elevation in the inferior and anterolateral leads as well as a prolonged QT interval (0.486 s) (Fig. 1A). Initial troponins at the time of admission were normal (0.023 ng/ml). Her pro-BNP was 103 pg/ml. Her erythrocyte sedimentation rate was 7 mm/h and her C-reactive protein was 1.34 mg/l.

**Fig. 2:**
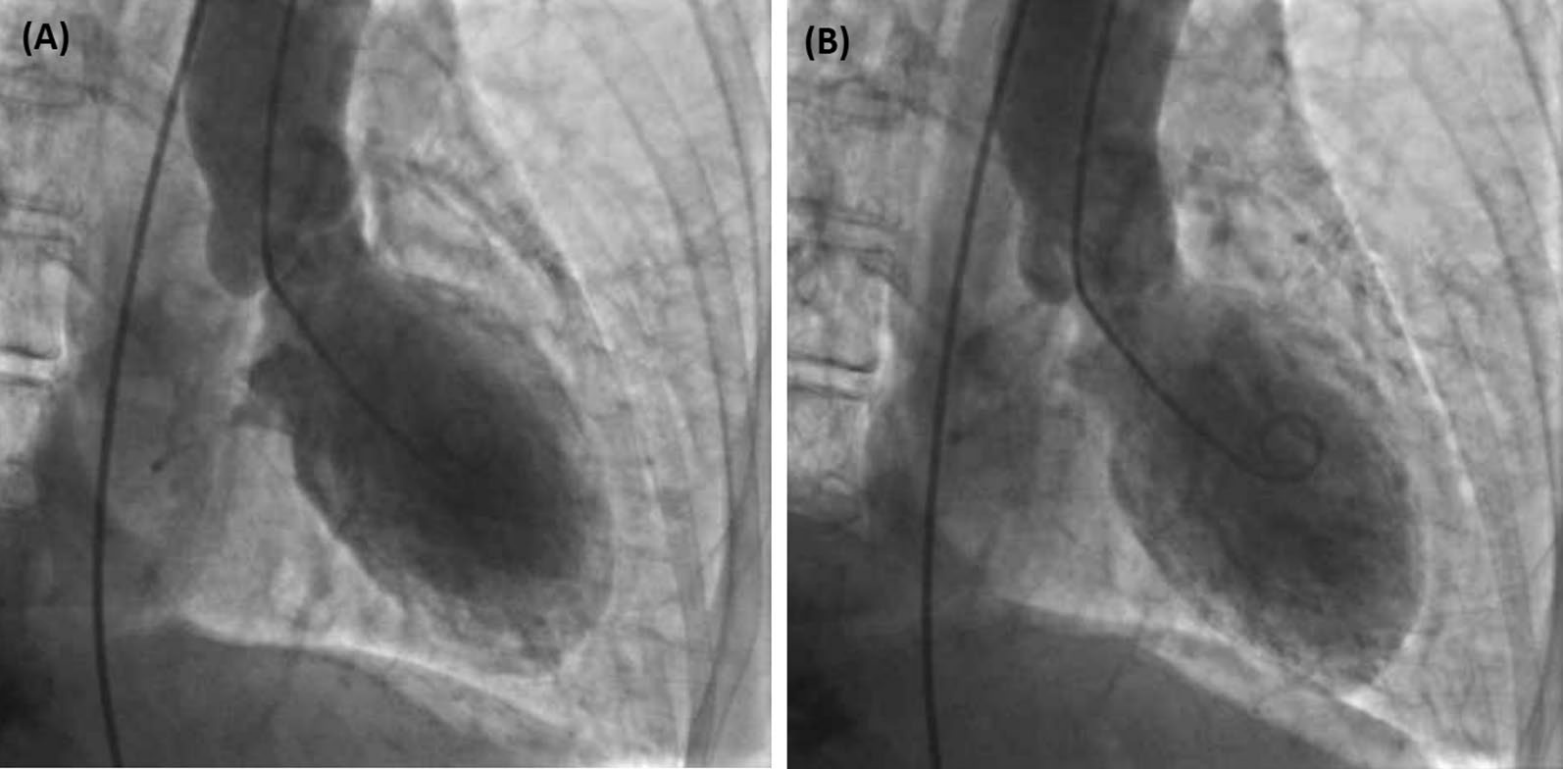
Left ventricular angiography in **A** diastole and **B** systole demonstrating akinesis of anterior wall, apex, and inferior wall in setting of non-obstructive coronary artery disease

Patient was transferred to critical care unit for further management, on BiPAP and was treated for acute asthma exacerbation. A repeat EKG obtained 1.5 hours later showed Left axis deviation but no significant changes from the initial EKG (Fig. 1B). Troponins were trended overnight and continued to rise but were moderately elevated (3.97 ng/ml and 4.65 ng/ml). An EKG the following morning 18 hours later showed a new onset Left bundle branch block (LBBB) (Fig. 1C). She came off the BiPAP and was transitioned to Nasal cannula, was feeling improved the morning of the Day 2. That evening, she started to deteriorate again requiring BiPAP. An echocardiogram (ECHO) was obtained at this time, which revealed an Ejection fraction (EF) of 24%, with severely depressed LV function but normal wall thickness and shape (Additional file 1: Fig. S1). A prior ECHO done eight months back showed an EF of 65%, without any regional wall motion abnormalities. Due to her worsening respiratory status, new EKG changes from that morning and increasing troponin, she was taken to the Cardiac Catherization lab urgently. Cardiac catheterization revealed normal coronary angiogram, an EF of 10%, and Left Ventricular End Diastolic Pressure of 35 mm Hg. A pro-BNP resulted that time was 20,242 pg/ml. Left ventriculogram showed akinetic apex, anterior and inferior walls with mild dilation of the left ventricle supporting the diagnosis of TCM (Fig. 2). An intra-aortic balloon pump (IABP) was placed for circulatory support, however vasopressors and inotropes were not required.

The patient was started on carvedilol and enalapril and she was monitored in the critical care unit until the IABP was removed. The rest of the hospital course was unremarkable on the medicine floors and she was discharged after being weaned off supplemental oxygen. She opted out of anticoagulation. Follow-up ECHO 9 weeks later revealed normal left ventricular function with EF of 59%.

## Discussion

We presented a case of TCM in a 68-year-old female presenting with asthma exacerbation. Within 24 hours of presentation, she developed a new onset LBBB on EKG and had mild troponin elevation. Her respiratory status improved the morning of the day 2, but then deteriorated again the night of day 2. An ECHO showing low EF, increasing troponin, continuing ST changes, and worsening respiratory status prompted an emergent cardiac catheterization for high-risk NSTEMI. The cardiac catheterization revealed findings that were highly suspicious of Takotsubo syndrome.

Currently, the diagnostic criteria adopted by the Heart Failure Association and European Society of Cardiology are the most widely used in the diagnosis of TCM. In addition to the above definition, criteria includes an absence of atherosclerotic disease that would explain the presentation, new and reversible EKG changes (ST elevation/depression, LBBB, and/or QTc prolongation), elevated Brain natriuretic peptide (BNP) or natriuretic-pro-BNP, slightly elevated troponins and recovery of the ventricular function on imaging at 3 to 6 months. The International Takotsubo Diagnostic Criteria has been recently proposed and is similar to the criteria put forth by the Heart Failure Association and European Society of Cardiology with the exception that infectious myocarditis must be ruled out [19]. Myocarditis requires a biopsy for a definitive diagnosis; however a biopsy for this purpose is not commonly done in our facility. Clinically, myocarditis presents with chest pain, which our patient did not report. Other findings suggestive of myocarditis include global dilation on ECHO, elevated inflammatory markers (ESR, C-reactive protein, etc.), and non-specific changes on EKG, which were not seen in our patient. A LBBB is associated with a worse prognosis in patients diagnosed with myocarditis.

Given its similarity in presentation to ACS, it is imperative to differentiate TCM from ACS. There are certain findings on EKG and imaging that can help differentiate TCM from ACS, although are not definitive. One finding on EKG that is 100% specific for TCM is ST elevation in AVR combined with ST elevation in more than 2–3 anteroseptal leads (V1-V6). This finding has been reported to have a positive predictive value of 100%, a negative predictive value of 52%, and a sensitivity of 12% [20]. Another finding that may suggest TCM as opposed to ACS is the differences in elevation of cardiac troponins. In TCM, troponins are typically elevated less than 10 ng/ml. BNP and natriuretic-BNP are typically much higher in TCM as compared to ACS. An ECHO may be able to demonstrate the classic apical ballooning form, however, it cannot be differentiated from ischemia involving the apex. Cardiac magnetic resonance imaging has also been used to differentiate TCM from ACS [21]. Delayed gadolinium enhancement, which helps to demonstrate myocardial edema, tends to match the area of akinesis in TCM during the acute phase of illness while it is more localized to the involved coronary artery in ACS.

The pathophysiology of asthma-induced TCM is not well understood. It is hypothesized that the asthma attack itself leads to excessive production of catecholamines and Neuropeptide Y, and it may trigger apical cardio-depression precipitating TCM [22]. However, it is not clear if elevated levels of these substrates are a reflection of the pathophysiology or if this is the cause. However, direct exposure of myocardium to catecholamine is deemed to be highly toxic and may cause cellular damage, altered cellular metabolism, and contraction band necrosis [23, 24]. Further, there is evidence to suggest that selective beta-2-agonists may induce apoptosis of cardiac myocytes due to production of reactive oxygen species [25, 26]. Other possible mechanisms proposed include the possibility of increased beta receptors in certain areas of the myocardium (9), changes in G-protein signaling from Gs (stimulatory) to Gi (inhibitory) leading to negative inotropic response (11), and in the event of increasing beta agonist use, the loss of selectivity of these drugs to lung tissue leading to vascular spasm in the myocardium (13).

The main complication of TCM is cardiogenic shock resulting in circulatory failure. The management is supportive, with severe cases requiring Mechanical Circulatory Support with Bridge to Recovery. It is important to determine if a left ventricular outflow tract obstruction (LVOTO) is present (as determined by ECHO). In the case of LVOTO, the obstruction must be relieved prior to treating the heart failure. Given risk of arrhythmias, continuous EKG monitoring is recommended for at least 48 h.

We performed a review of literature and found 11 other cases that describe asthma exacerbation as a trigger for TCM (Table 1). Six out of the 11 cases we reviewed were post-menopausal females. None of the cases, including ours, exhibited the specific EKG finding for this syndrome. However, there is only one other case to our knowledge that presented with a new LBBB [13]. All cases were treated with a loading dose of steroids and inhaled bronchodilators for management of asthma exacerbation. In our study, additionally, repeated doses of magnesium sulfate were used for the management of status asthmaticus. Other notable treatments used in prior reports included combinations of epinephrine/ketamine and aminophylline. Our case presented with similar troponin elevations, ECHO findings, and cardiac catheterization findings as seen in previous reports.

We suspect that our case of TCM was induced by the physiological stress of an asthma exacerbation or possibly by the administration of beta agonistic drugs. However, the former seems more likely as our patient had EKG changes (ST Elevations in the inferior and anterior–lateral leads) on admission that were not present on a prior EKG and she did not report any chronic and excessive use of inhaled beta agonists. We came to the conclusion that her asthma exacerbation lead to the development of TCM immediately on or prior to admission. The new LBBB on day 2 prompted the cardiac catheterization. The ST elevations and LBBB resolved completely after the catheterization.

## Conclusions

This case highlights the importance of maintaining a high suspicion for TCM in post-menopausal women with asthma exacerbations, who have persistent symptoms despite treatment with bronchodilators and steroids, even in the absence of an emotional trigger.

## Supplementary Information

**Additional file 1: Fig. S1**. Echocardiogram with demonstration of hypokinesis of entire septal area, apex, and lateral areas. On the base contracts (arrow).

## Data Availability

Data sharing is not applicable to this article as no datasets were generated or analyzed during the current study.

## Abbreviations

TCM: Takotsubo cardiomyopathy
LV: Left ventricle
ACS: Acute coronary syndromes
EKG: Electrocardiogram
LBBB: Left bundle branch block
ECHO: Echocardiogram
EF: Ejection fraction
IABP: Intra-aortic balloon pump
BNP: Brain natriuretic peptide
LVOTO: Left ventricular outflow tract obstruction
RBBB: Right bundle branch block
WMA: Wall motion abnormalities
TWI: T-wave inversion
