# Supplementary material for: Takotsubo cardiomyopathy in a female presenting with status asthmaticus: a case report and review of literature
Source: Egypt Heart J. 2022 Oct 1;74:72. doi: 10.1186/s43044-022-00310-9 (PMC9526768; doi:10.1186/s43044-022-00310-9)

Supplementary Image 1- Echocardiogram with demonstration of hypokinesis of entire septal area, apex, and lateral areas. On the base contracts (arrow).

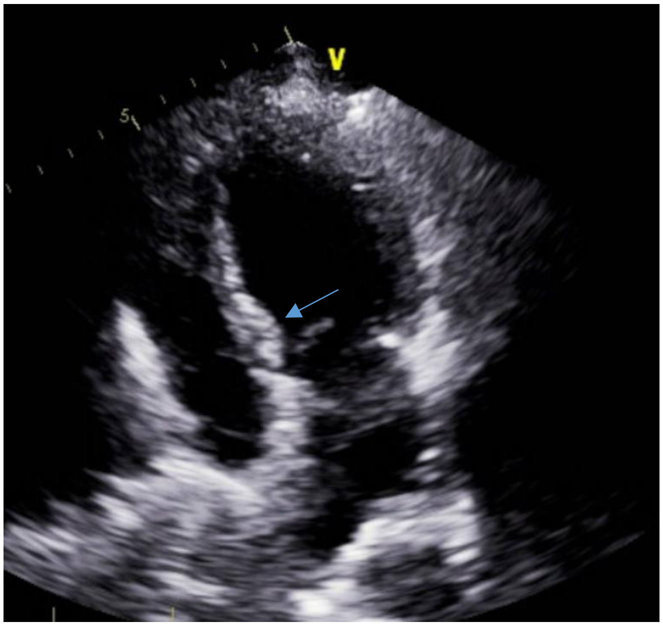

Supplement: Supplementary file 1 — Additional file 1: Fig. S1. Echocardiogram with demonstration of hypokinesis of entire septal area, apex, and lateral areas. On the base contracts (arrow). [file 43044_2022_310_MOESM1_ESM.pdf]
